# Medical Jurisprudence of Insanity
*Lectures on the Medical Jurisprudence of Insanity, delivered in the Medical School of King's College, Aberdeen. By Robert Jamieson, M.D., Lecturer on Medical Jurisprudence in the University. London, Wilson. 1851.


**Published:** 1851-04-01

**Authors:** 


### Art. III.—MEDICAL JURISPRUDENCE OF INSANITY.*

The discoveries and improvements which have taken place in the theory and practice of medicine, have necessarily opened up new paths, and led to the recognition of different branches of the profession. Instead of being studied collectively, we now find intelligent physicians select certain diseases as specialities, which subdivision of labour has been attended with the happiest results. If Laennec had not devoted his attention almost exclusively to diseases of the lungs and heart, the stethoscope, which has led to so many brilliant discoveries never would have been introduced into practice; if Dr. Bright had not specially studied renal pathology, he would not have discovered the nature of a disease which was previously unknown to the profession. In like manner we are sanguine enough to believe that by making a speciality of the study of the brain, in connexion with various forms of mental derangement, light will eventually be thrown upon the pathology of their embarrassing diseases. The different branches of medical science thus become eventually detached from each other, and each separately identified so as to form a distinct object of investigation. It becomes, therefore, ever and anon, necessary to add a new course of lectures to the curriculum of medical study, and occasionally to endow new professorships. The history, however, of every university will show that the patrons—whether their patronage be vested in the crown or in municipal authorities — have always evinced extreme reluctance to recognise any new or additional branch of education. Not many years ago, chemistry and materia medica were jumbled together, as in the days of Paracelsus; until very lately in some universities, anatomy and surgery were taught from the same chair; and it is a matter of notoriety, that whenever any new course of lectures has been suggested within the walls of a university, the proposition has always been met with strenuous opposition. But the stream of intellectual progression must flow onwards, acquiring force as it deepens and widens, until all such obstacles are borne down before it. Who now doubts the expediency of a professorship of medical jurisprudence, military surgery, or pathology? Had there been no chair of moral philosophy in the north, the world never would have been illumined by the lectures and writings of such men as Reid, Dugald Stewart, Brown, &c.; and at the present moment it is, we conceive, deeply to be deplored that in our

* Lectures on the Medical Jurisprudence of Insanity, delivered in the Medical School of King's College, Aberdeen. By Robert Jamieson, M.D., Lecturer on Medical Jurisprudence in the University. London, Wilson. 1851.

English schools of medicine the study of mental philosophy is wholly neglected. We do not wish to go back to the scholastic ages, but we are satisfied that some knowledge of dialectics would be useful to every medical man; and however much the study of psychology may be scoffed at and ridiculed by men who talk blindly and ignorantly upon matters they do not comprehend, we believe that no physician can be competent to discharge his duties by the bed-side, who has not paid some attention to the constitution of the human mind. Unhappily, insanity—however varied be the forms it may assume—is a disease so frequently met with, and so distinct in its own nature, that it can no longer be denied a place among those diseases which from their peculiar and uniform characteristics are entitled to the rank of specialities.

In that dark age when the lunatic was fettered like a convict, and chained miserably against a wall or pillar, the fact of the disease itself being of a character which required medical and moral treatment was entirely overlooked, and an error equally palpable and revolting is constantly committed by those who evince a disposition to underrate and decry this branch of the profession. The pathology of the brain is assuredly as much entitled to consideration as that of other vital organs, which indeed play a less important part in the animal economy; and there can be no reason why lectures should not be regularly given in our medical schools upon a subject of so much importance. We hear, it is true, of occasional lectures upon insanity; but we maintain that a course of lectures upon this subject ought to be regularly delivered in every medical school. Such a course has, for the first time, been just delivered at the King's College, Aberdeen, by Dr. Jamieson, the professor of medical jurisprudence in that University; they have been published in our respected cotemporary, the "Medical Gazette," and now appear before us in a distinct form. The title-page would induce us to expect that Dr. Jamieson confined his observations to the medical jurisprudence of insanity; but instead of this, he has treated the subject generally, and described the disease in all its different phases and relations.

In endeavouring to give a brief abstract of these lectures, we shall select the most salient points of interest, premising only that our limits necessarily compel us to pass over many important and valuable observations. The views which Dr. Jamieson entertains upon the pathology of insanity, would appear to be intermediate between those which are materialistic and spiritual.

"Some would have it (he tells us) a disease of the body in which metaphysics are useless; others, a disease of the mind to which physic is as inappropriate. Still we find it curable, sometimes by moral treatment, sometimes by medical, but far more usually by a judicious combination of the two, and almost never remedied by unassisted nature. It is not a purely corporeal disease, like one of the neuroses; it is not a nervous affection merely, but a neurosis and something more; neither is it purely a mental affection, like error or vice. Both mind and body are at fault. According to the views of Feuchtersleben, it is their relation that is diseased; of the body to the mind, so that perception is morbid; of the mind to the body, so that volition is disordered. From which, then, does that disturbed relation proceed which, when established, becomes reciprocal? If from the organism, it is a physical disease; if from the mind, a mental one, although not to be defined. Were I to dogmatise (adds Dr. Jamieson) on this abstruse matter, I would say that there are two kinds of insanity—one a mental disease, the other a bodily one, both acquiring the psychosomatic character. There is a form of insanity, produced by mental causes, in which the physical symptoms appear secondarily, which is sometimes curable by moral means, and which, if uncured, either leaves no traces behind, or a morbid anatomy, which is its result, and in no case its cause. The other proceeds from physical derangement, occasions sympathetic mental aberration, disappearing with its cause, and leaving no traceable pathology."

Notwithstanding this twofold pathological view, Dr. Jamieson leans to the theory of relation propounded by Feuchtersleben.

"I repeat (he observes, emphatically,) that insanity is not, strictly speaking, to be termed either a bodily or a mental disease; that it is a disturbed reciprocal relation of mind and body; but that, in its origin, it is sometimes a mental, sometimes a bodily disorder."

The emotional features of insanity—selfishness, suspicion, extreme timidity, apathetic listlessness—as characteristics of the impending or already confirmed state of mental disease, are truthfully and graphically described. They are familiar to all who have had any experience in the treatment of the insane. We proceed, therefore to develop the psychological views which Dr. Jamieson has propounded. The intellectual condition of lunatics—the true mental pathology of the disease—demands, indeed, the most careful investigation. In the analysis of the human mind, Dr. Jamieson recognises the existence of thought and *will* as essentially constituting mental consciousness. "The first (thought) represents that unfailing train of ideas which have their origin in an accidental series of external impressions on the senses; or which, if not *ab externo*, are connected together by certain laws of association. They consist of instinctive inclinations, emotional feelings, intellectual perceptions, and primitive rational intuitions. The second (will) represents the consciousness which we have of a certain amount of power in controlling or affecting the succession of our thoughts. This voluntary faculty does not create ideas, it merely selects, detains, and operates upon any particular idea of the many that crowd upon the mind. It is by this relation of our will to thought, that thought becomes thinking; desire, action; and abstract mind, personality. The agency of the will is apparent in both processes, as attention, judgment, and recollection; which are, in truth, voluntary contemplation, alternate and retrospective; but indeed the voluntary faculty is the essence of all intellectual operations whatever." These are Dr. Jamieson's views, but we must confess that we are not prepared to admit that the will plays so omnipotent a *rôle* in all the phenomena of mental manifestation, or that it is even essential to the existence of mental consciousness. Let us, however, follow Dr. Jamieson's application of this theory to the state of the mind in insanity.

"In the various forms (he continues) of intellectual unsoundness, both these principles—thought and will—seem in every case to be affected, though it would be more strictly correct to say that it is the relation of the latter to the former that is disturbed. The succession of ideas is more rapid than natural in maniacal excitement; more slow than natural in melancholia, and uncontrolled by its natural laws in dementia.

"The voluntary power over trains of thought is, in all cases, defective. In mania it seems overpowered by the force and rapidity of the current; in melancholia, enfeebled by the one vast and gloomy image that has dammed up the whole channel of thought; and, in dementia, permanently impaired. Such a condition of mind may occasion diseased manifestations of any or all of the intellectual processes, and it would not be difficult to select examples of disordered sensation, perception, attention, memory, conception, and so forth, occurring in lunatics; but the defect which is most characteristic of insanity is, as I have formerly stated, an inability to exercise the faculty of comparison, so as to evolve judgment upon one or more subjects, that incapacity being a direct consequence of impaired voluntary power over thought. In addition to such other evidence of disease as misinterpreted sensations and conceptions, riveted or distracted attention, and wayward and irresponsive memory, there is this distinct impracticability of judgment before there can exist an insane delusion."

There can be no doubt that, in all cases of insanity, the powers of comparison and judgment must be impaired; but we would put it to Dr. Jamieson whether the lesion of volition ought not to be considered as a form of insanity rather than as a predominating cause of every variety of the disease. In many cases of monomania, we have no evidence of the faculty of volition being impaired; neither do we discover it in many cases of melancholia. Accordingly, Heinroth, in his classification, enumerates three kinds of insanity: first, insanity affecting the moral disposition; second, insanity affecting the understanding; and, third, insanity affecting *voluntary* power, the propensities, the *will.*

Dr. Jamieson proceeds next to consider the origin of delusions in violent mental impressions which produce a stronger hold upon the belief than any physical sensation could convey. He observes, very truly, that a lunatic believes in his delusion more firmly than he ever did in the truth of any real object of sense. The possibility of being deceived he cannot admit; he will sooner admit all other accredited realities to be deceptive. Those senses which act soundly enough have no power to correct the error. Sir Walter Scott relates the case of a poor lunatic in the Edinburgh Infirmary who fancied that he was living in great state and splendour in a mansion of his own, his only unhappiness being that all the dainties with which his table was supplied had the taste of porridge. The palate acted an honest part, but its appeal to the judgment was quite ineffective. Any degree of doubt which may arise in an insane person's mind regarding the realities of his fancy is of a peculiar sort, and exerts no influence, or only one of the most transient and feeble kind. "I had a species of doubt," says a recovered maniac describing what were his feelings—"I had a species of doubt, but no one who has not been deranged can understand how dreadfully true a lunatic's insane imaginations appear to him—how slight his sane doubts." The definite character of those delusions which occur in monomania and their marvellous persistency, Dr. Jamieson dwells upon at some length, interspersing his observations with many illustrative facts which will be read with interest. The endeavour which insane persons sometimes make to conceal their delusion, induces Dr. Jamieson to remark that such attempts are never successful, for that the delusion existing in the mind will always in some measure be expressed in the habits and conduct of the patient. Nevertheless, the insane will often appreciate the motives which ought to lead them to hide their aberration, and they are well aware of the subjects on which it is necessary to exercise concealment, in order to pass muster as sound in mind; but for all this, they seldom or never succeed in concealing their delusion when they are directly questioned regarding it. The most that a lunatic, desirous of passing as sane, can in general effect, is not of himself to introduce the subject upon which his judgment is alienated; but when he is expressly catechised upon it, he either refuses to answer any questions regarding it, which is to him a very great effort, or he certainly stumbles in his replies, and makes evident the unsoundness of his understanding. An insane lady had so far imposed upon a philanthropic visitor of the asylum in which she was confined, as to lead to a private and influential representation to the sheriff that she was unnecessarily and unjustly deprived of her freedom. The sheriff accordingly called unexpectedly at the hospital and had a private audience in order to satisfy himself of her condition. She answered every question in so rational a style as to afford no apparent grounds upon which her liberation should be refused. Upon consulting the case book, however, he found that she was said to entertain the belief that she was the Duchess of Wellington, or at times the Baroness Rothschild. Having obtained the key to her mystery, he held a second conversation with her, when, in spite of her anxiety to impose upon one whom she knew to have the power of terminating her confinement, she exhibited such evident insanity, that he left her apartment holding up his hands in amazement. She could not resist a direct interrogatory upon her rank of duchess. Upon this subject the following practical observations deserve particular attention:—

"Delusions," says Dr. Jamieson, "are liable to be modified by such circumstances as sex, age, temperament, education, and bodily disorder. Depressing fancies are much more common than those which are called exciting. In 287 instances of monomania I found that the low (or depressing) delusions amounted to 168; the high (or exciting) delusions to 76; and those which were of an indifferent order to 43. High delusions were more frequent in the male than in the female sex. The only temperament in which they relatively predominated over those of a low type, was the sanguine: in all the others, particularly in the lymphatic or deteriorated sanguine depressing imaginations, were the more numerous. High delusions have a tendency to be transformed into those of a depressing character. Education modifies the nature, but more strikingly the expression of insane convictions. An intelligent person, for instance, will not be found styling himself the 'fourth person of the Trinity;' nor will an educated female write her name 'Margaret Rex.' The sensations which compel an ignorant lunatic to express his belief that he has been bewitched, would, in one above superstition, lead to the notion that he had been brought under the influence of some kind of magnetic or mesmeric agency. Bodily disorder may, there is some reason to think, produce a modifying effect. Diseases of the lungs or of the encephalon are more likely to be conjoined with delusions of a high cast than those of the kidneys or heart. Almost all cases of insanity which threaten apoplexy or paralysis, are associated with high delusions. In those cases, also, which are complicated with the peculiar paralysis of the insane—an insidious general palsy—exciting fancies are nearly universally observable. Such persons, while they are scarcely intelligible from defective articulation, while they are staggering with extreme difficulty from one chair to another, still have a shattered physiognomy of happiness. They are in a paradise of excitement, leading armies over Alps, or dispensing the riches of India. When death is making daily approaches upon them, they are in a state of miraculous health; and when they are in his very clutches, they are in the arms of victory, or the glories of Solomon's Temple."

In a note appended to these observations Dr. Jamieson gives the following ratios between different temperaments and high and low mental delusions:—

| | High Delusions. | Low Delusions. |
| --- | --- | --- |
| | Per Cent. | Per Cent. |
| Aggregate Cases | 31 | 69 |
| Nervous Temperament | 30 | 70 |
| Bilious ditto | 26 | 74 |
| Sanguine ditto | 60 | 40 |
| Lymphatic | 8 | 92 |

The views which lunatics take of their insane companions has often struck us not only as being curious, but as being pathognomonic of particular morbid states of thought and feeling. It frequently happens, especially in cases of extreme imbecility, that the patient takes no cognizance of his own state, nor discovers any thing extraordinary in the conduct of those around him; but, in many instances, they are perfectly conscious of the very forms of insanity which surround them, and will frequently jeer and jibe at each other for the delusions and peculiarities they entertain. We have frequently heard one lunatic say of another, "Ah! he's mad! He'll never get out of this place." There is, however, one circumstance connected with their mode of reasoning which should be particularly noticed. They are often perfectly sensible of their real position, argue shrewdly upon the legality of their case, and have a full knowledge of their irresponsibility in the eye of the law. The observations of Dr. Jamieson upon this subject are extremely pertinent. A lunatic will seek to obtain his freedom by bribing his attendant with a promissory note for a considerable amount; and in a private communication to one of his friends will frankly confess what he intends to do, remarking that, of course, there is no danger of loss to be apprehended, for that his position incapacitates him from being lawfully a party in any agreement. This might be pleaded by the patient's curators or his heirs, but not at any time by himself, for it is a principle in law that no man shall stultify his own acts. Not long ago, says Dr. Jamieson, under my own observation, an insane patient made the attempt to destroy the life of his attendant, who was at the time in a stooping position, by suddenly snatching up a spade and aiming a blow at his head. Manslaughter, he said, was of little consequence to him, for no madman is punishable by law. Such speeches, Dr. Jamieson adds, do not indicate that the individual is aware of and believes in his own madness, but only that he knows he is accounted insane. No person, therefore, who falls suddenly into a state of mental derangement, and commits a deed of violence, can be supposed to do so, because he knows that he will be exempted from punishment on account of the state of his mind. A criminal lunatic does not believe in his own insanity at the time that it is pleaded for him by his counsel. He may become aware of his legal position only when experience has taught him how his conduct is judged by the world.

The intellectual disorder of lunatic patients leads them, however, not unfrequently to commit acts of the most singular and inexplicable extravagance, which are frequently suggested by some delusion which it is difficult to conjecture. Dr. Jamieson mentions the case of an excited maniac, who spent his time mostly in turning somersets on the ground; certainly an absurd and dangerous occupation for a person past his boyhood, whose business was the grave study of theology. There was no alleged or apparent motive, and little other indication of delusion. It, however, upon explanation, turned out that this was done at the instigation of a voice in his head, as being the mode of worship most acceptable to heaven. Another lunatic manifested a strange propensity to climb up the interior of chimneys, to the great danger of his life; in truth, he narrowly escaped suffocation and burning; he was for a time constantly bent upon this as the great aim of his existence. It was an action which no one could pretend to understand, unless as a peculiar kind of suicidal intention, which, however, it was not. He told Dr. Jamieson, after his recovery, that he believed that the only way in which it would be possible to persuade mankind that he was truly the Emperor of Russia, as he then supposed himself to be, was by ascending to the roof of the house in that manner. He was a silent lunatic, whose actions were the source of much care and anxiety, and whose delusions could never be surmised. A maniac, during his paroxysms, amongst other acts of destructiveness used to occasion much trouble by tearing his shirt and bedding into shreds during the night. After recovery, he accounted for his so doing in this way. He said that he was perfectly aware of what he was about, but that he had no idea then that it was anything wrong; on the contrary, he did it more to please others than himself, and actually believed that it was a very meritorious action, which would do some vague kind of good to himself and others, provided he succeeded in tearing the articles into certain determined shapes. He was very much disappointed to find, when visited in the morning, that he was blamed instead of being praised by the attendant; and this he attributed, not to having torn his bedding, but to his not having succeeded in making the fragments of the proper shape. In the published narrative of one who experienced and recovered from a lengthened attack of mental derangement, the same absence of malice is prominently represented. "I knew no malice," he says, "no vice. I imagined the keepers all loved me and were deeply interested in the salvation of my soul; and I imagined too that I loved them dearly. Yet I wrestled with them, and offered to do so with others, and struck many hard blows sometimes, as one informed me, making it difficult for three men to control me; yet whenever I did this I was commanded that they wished me to do so to prove my faith and courage, but that they were commanded to prove both till they were satisfied of my sincerity. It was a great delight to me to get my hand at liberty, even for a moment, and the first use I usually made of it was to strike the keeper who untied me; directed by my spirits to do so as the return he deserved, above all things else, because he knew I was proving my gratitude to the Lord Jehovah at the risk of being struck myself."

Dr. Jamieson next passes under review the different kind of insane impulses which prompt to the commission of suicide and homicidal acts. He distinguished three kinds of suicide. Self murder, or that voluntary rational suicide which is implied in the technical phraseology of *felo de se*. Insane suicide, resulting obviously from insanity, and suicidal moral insanity. In reply to the question, when is suicide to be considered as the act of insanity? Dr. Jamieson answers:

"Self-destruction ought to be held as insanity when it is the deed of one hereditarily predisposed to mental disease, or to death by suicide, particularly if the predisposition is inherited from the parent of the same sex, as this gives increase and force to congenital tendencies. Dr. Gall, Dr. Barrow, and other writers, have given striking examples of suicide occurring in three or four consecutive generations of a family, the result undoubtedly of inherited qualities, and not to be accounted for on the mere ground of suggestive example and vicious imitation. Self destruction is to be deemed insanity, also, in all those cases in which the circumstances of the individual were not such as to develop any of those motives which occur in cases of self murder or *felo de se;* so, likewise, in every instance in which it is to be ascribed to mere imitation, or to the fascination of suggestive opportunity. The mode of committing the action may itself be indicative of disease. If little could be inferred from the single fact of a person hanging, drowning, poisoning, or pistolling himself, there could certainly be no doubt of the state of mind of one who chose to throw himself into a furnace, to starve himself to death, to crucify himself, or kill himself by self-mutilation. Suicide amounts to proof of insanity when committed by people labouring under certain diseases; as pellagra, an endemic skin disease, which, according to Professor Tomassini, occasions often an irresistible impulse to self-annihilation; hypochondriasis, paralysis, hysteria, epilepsy, extreme bodily pain, uterine derangement, spermatorrhœa, nostalgia, intoxication, &c., all of which are efficient causes of mental diseases, and many of them states which are either transitial to, or premonitory of, insanity. Any of those pathological appearances which are more peculiarly connected with mental alienation, if found in the body of a suicide, ought to lead us to the inference that the individual laboured under mental disease; and, indeed, in the present state of our knowledge, if we can distinctly make out that he suffered from bad health, the violent death should be ascribed to sympathetic cerebral disturbance. In short, suicide is presumptive of insanity, and ought to be held probative in every case in which the recognised causes of self-murder were not clearly present."

We have elsewhere advocated this opinion, and pointed out the simple fact that, speaking generally, in cases of suicide the bodily health suffers before the mind reconciles itself to the appalling act of self-destruction.

The medical jurisprudence of insanity, as we have premised, occupies only a small portion of these lectures; but that portion is perhaps the most valuable. In commenting upon the criminal responsibility of the insane, Dr. Jamieson observes:

"Before a person can be deemed responsible for his actions, he must not merely have the power of *distinguishing* right from wrong; but the power of *choosing* right from wrong; a criminal being properly punishable, not because he knew good from evil, but because he voluntarily did the evil, having the power to choose the good. If a special test of insanity were to be insisted upon, the power of self-control, as being the true index of responsibility, would seem to be better than that of the integrity, either of consciousness or conscience. Had the lunatic, at the time of committing the deed, a knowledge that it was a criminal one, and such a control over his actions as might, if exerted, have hindered him from committing it? Most lunatics have an abstract knowledge that right is right, and wrong, wrong; as much of it as should keep them from being guilty of unlawful deeds, were but such knowledge sufficient for that end; but the voluntary power over action and thought is in every case impaired. I do not say that free agency is annihilated; this were untrue: there could then be no moral treatment of insanity; but it is much limited and over-ruled by various insane motives. Lunatics have that amount of free will which it is philosophical, charitable, and advantageous to recognise for their benefit; but, at the same time, such a defect of free agency as makes the full burden of responsibility to imperfect human legislation a discreditable and unjust oppression. Many of them may be fully accountable *in foro conscientiæ;* but in all other courts, if the insanity be apparent, the defect of self-control should be presumed to exist; and the individual condemned to restraint, or if considered liable to punishment, subjected to only a mitigated penalty."

These and Dr. Jamieson's other views are characterized by feelings of humanity, which ought to modify our judgment upon all acts of insanity; and we trust that he will every session deliver a similar course of lectures. They are calculated to be of great advantage both to the profession and the public, and we could fain wish that in this respect the medical schools of other universities would follow the example of the King's College at Aberdeen.

